# Efficacy and safety of regional citrate anticoagulation using calcium-containing replacement solution in different modalities of continuous renal replacement therapy: a randomized controlled trial

**DOI:** 10.1186/s12882-025-04565-7

**Published:** 2025-11-06

**Authors:** Peiyun Li, Mingpeng Li, Wanhong Yin, Zhifeng Zhou, Ling Zhang

**Affiliations:** 1 Department of Nephrology, Kidney Research Institute, West China Hospital of Sichuan University, Chengdu, 610041 China; 2 Department of Nephrology, The People’s Hospital of Jianyang City, Jianyang, Sichuan China; 3 Department of Critical Care Medicine, West China Hospital of Sichuan University, Chengdu, 610041 China

**Keywords:** Continuous renal replacement therapy, Regional citrate anticoagulation, Calcium-containing replacement solution, Lifespan of extracorporeal circuit, Safety

## Abstract

**Objective:**

Regional citrate anticoagulation (RCA) is recommended as the first choice of anticoagulation strategies in continuous renal replacement therapy (CRRT). However, when using calcium-containing replacement fluid, whether RCA can achieve sufficient anticoagulant effect among different CRRT modalities remains unclear.

**Materials and methods:**

In this open-label, three-arm, randomized trial, AKI patients receiving RCA-CRRT with calcium-containing replacement fluids were randomized to continuous veno-venous hemofiltration (CVVH), continuous veno-venous hemodialysis (CVVHD) and continuous veno-venous hemodiafiltration (CVVHDF) groups from 2022 to 2023. The primary outcomes were circuit lifespan and early circuit failure. Secondary endpoints included the safety of the three groups and 30-day all-cause mortality.

**Results:**

A total of 121 patients were enrolled, with 40 in CVVH group, 40 in CVVHD group, and 41 in CVVHDF group. The circuit lifespan in CVVHDF group (70 h, IQR 65–72) was significantly longer than that in CVVHD (47 h, IQR 31.5–54) and CVVH group (64 h, IQR 46–71) (*P* < 0.001). Meanwhile, 27 circuits remained patent for 72 h (CVVH: 10/40, 25%; CVVHD: 1/40, 2.5%; CVVHDF: 16/41, 39.02%; *P* = 0.003). A significantly lower incidence of severe venous chamber thrombosis was observed in the CVVHDF group (2.4%, 1/41) relative to the CVVH (5%, 2/40) and CVVHD (50%, 20/40) groups (*P* < 0.001). Furthermore, early circuit failure (lifespan < 48 h) was observed in 35 circuits, occurring significantly more frequently in the CVVHD group (52.5%, 21/40) than in either the CVVH (22.5%, 9/40) or CVVHDF (12.2%, 5/41) groups (*P* = 0.004).

**Conclusion:**

Our findings suggested that RCA-CRRT with calcium-containing replacement solution appeared to be safe and effective, with CVVHDF showing potentially prolonged filter survival compared to alternative modalities, which might support its consideration as a possible option in clinical practice.

**Trial registration:**

This study was registered in the Chinese Clinical Trial Registry (www.chictr.org.cn) under the registration number ChiCTR2200061065 on 15 June 2022.

**Supplementary Information:**

The online version contains supplementary material available at 10.1186/s12882-025-04565-7.

## Introduction

Continuous renal replacement therapy (CRRT) has been widely applied in the treatment of critically ill patients with acute kidney injury (AKI) [1]. The treatment modalities of CRRT mainly include continuous veno-venous hemofiltration (CVVH), continuous veno-venous hemodiafiltration (CVVHDF), and continuous veno-venous hemodialysis (CVVHD). However, early clotting of the extracorporeal circuit can reduce solute clearance and ultrafiltration, thereby diminishing the efficacy of CRRT. Therefore, safe and effective anticoagulation, which prolongs the lifespan of the extracorporeal circuit, is crucial for the success of CRRT. Regional citrate anticoagulation (RCA) outperforms unfractionated heparin and low-molecular-weight heparin in prolonging extracorporeal circuit lifespan and reducing bleeding risk during CRRT [2, 3]. It is the preferred strategy for anticoagulation in patients with AKI receiving CRRT according to the Kidney Disease: Improving Global Outcomes (KDIGO) guidelines [4]. In conventional RCA protocol, the use of calcium-free replacement and dialysate solutions is universally recommended. This approach is theoretically grounded in the prevention of supplementary calcium ions from binding with citrate, thereby preserving the anticoagulation potency of citrate. However, the utilization of calcium-free solutions necessitates complex formulation and vigilant monitoring. Following our previous study reporting a simplified RCA-CVVH protocol using calcium-containing replacement fluid in 2013, RCA has been more widely adopted due to its simplicity and safety [5]. Previous studies have demonstrated that RCA-CRRT using calcium-containing replacement fluid is both safe and effective and reduces the clinical workload [6–8]. Whether RCA provides consistent efficacy and safety with calcium-containing replacement fluids across different CRRT modalities remains uncertain. We therefore conducted a RCT to compare circuit longevity and safety outcomes among these modalities.

## Materials and methods

### Study design

**Fig. 1:**
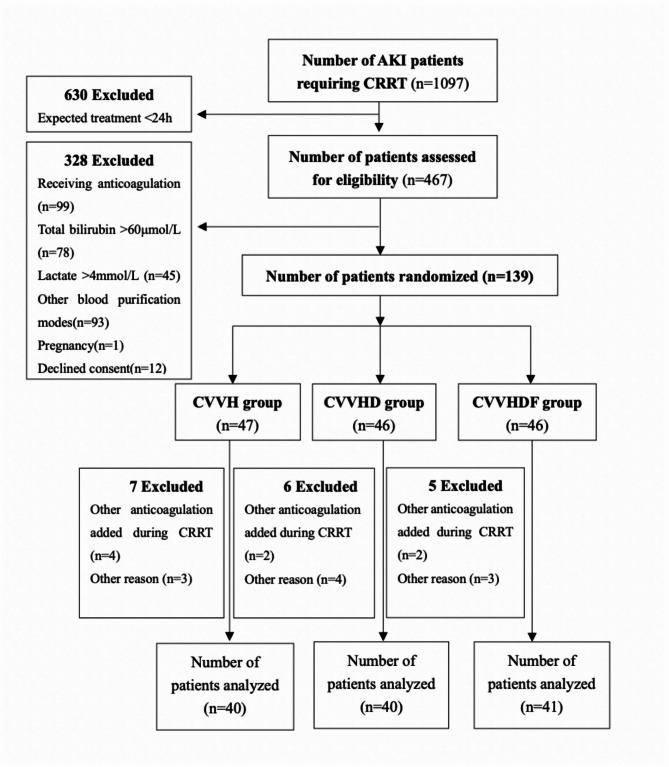
Flow diagram of the study

A prospective open-label, three-arm, parallel-group RCT was performed in intensive care unit (ICU) of West China Hospital of Sichuan University from June 2022 to May 2023. The conduct of the study will adhere to the Declaration of Helsinki (version Fortaleza, 2010). Informed consent was obtained from patients or patients’ legal representatives (Fig. 1).

### Patient and public involvement

This study did not engage patients or the public in its design, conduct, or dissemination processes; results will be shared with the public as necessary.

### Participants

Our study included adult individuals aged 18 or above who were identified with AKI according to the KDIGO criteria [9]. Initiation of CRRT was based on the RENAL study criteria: oliguria unresponsive to fluid resuscitation (urine output < 100 ml in 6 h), serum potassium > 6.5 mmol/L, severe acidemia (pH < 7.2), serum urea nitrogen > 70 mg/dl (25 mmol/L), serum creatinine > 3.4 mg/dl (300 µmol/L), or clinically significant organ edema [10]. Exclusion criteria included a history of citrate allergy, pregnancy, receipt of other anticoagulation within 3 days before or during CRRT, serum total bilirubin > 60 µmol/L, lactate > 4 mmol/L, or concurrent use of other blood purification therapies (e.g., plasma exchange, hemoperfusion). Patients were withdrawn if they used other anticoagulants during the study, requested to discontinue participation, or had the extracorporeal circuit discontinued before 72 h due to transfer, surgery, or discharge. Participants demonstrating citrate accumulation [11] (defined as total calcium/ionized calcium ratio > 2.5) or refractory hypocalcemia were permitted to discontinue the study and transitioned to alternative anticoagulation regimens (e.g., low-molecular-weight heparin or heparin-free protocols). Early withdrawal requests triggered immediate protocol termination with subsequent anticoagulation strategy adjustments per clinical standards.

### Intervention and CRRT protocol

**Fig. 2:**
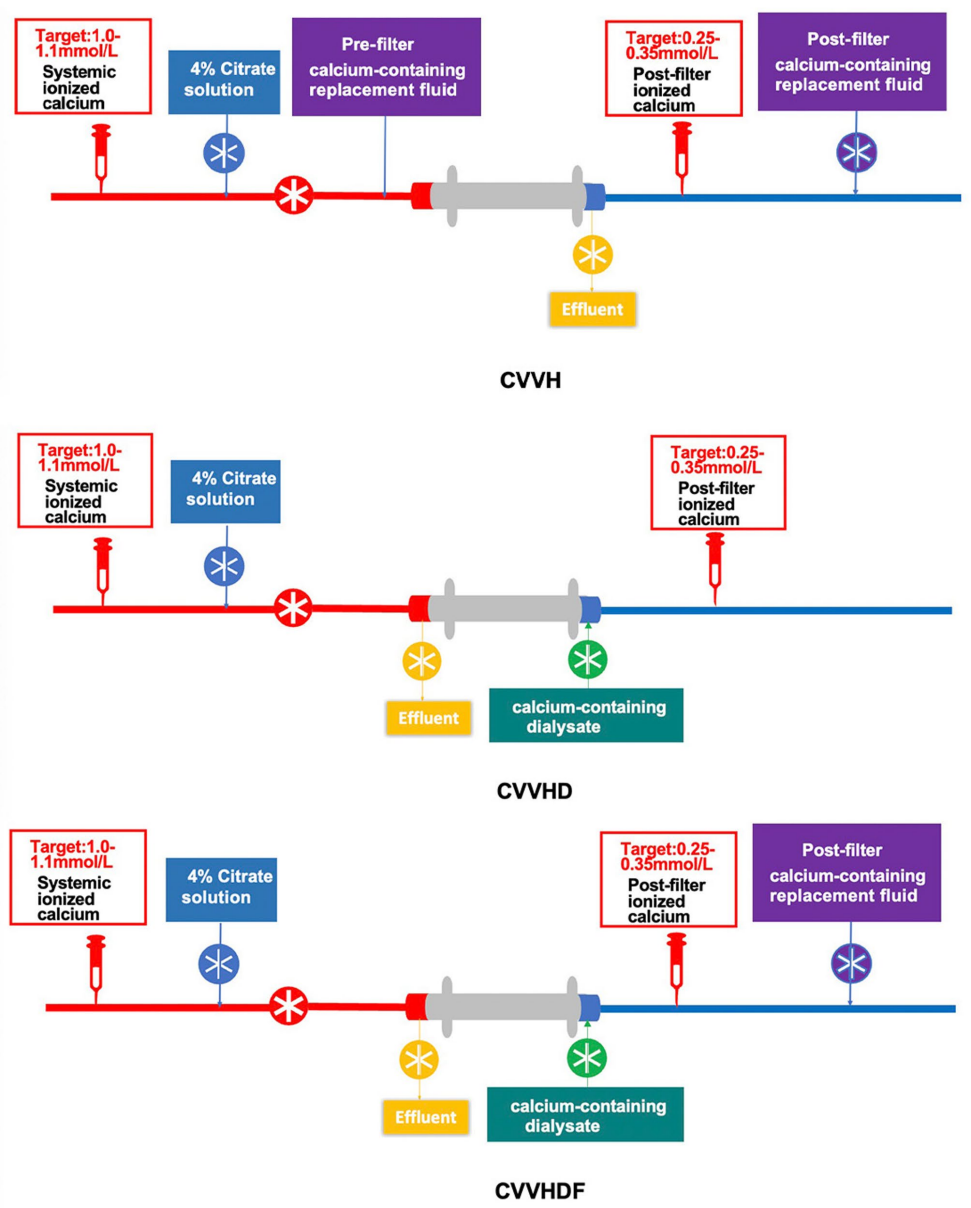
Schematic diagram of different CRRT modalities. CVVH, continuous veno-venous hemofiltration; CVVHD, continuous veno-venous hemodialysis; CVVHDF, continuous veno-venous hemodiafiltration

All CRRT procedures were performed by nurses with demonstrated clinical expertise in extracorporeal blood purification techniques. All CRRT devices used were Prismaflex V8.0 (Baxter Investment Co., Ltd), equipped with AN69-ST150 hemofilters (Baxter Investment Co., Ltd). Vascular access was provided by the insertion of a double lumen catheter into either the femoral vein (Baxter Investment Co., Ltd, GDHK-1325, 13Fr, 250 mm) or internal jugular vein (Baxter Investment Co., Ltd, GDHK-1215, 12Fr, 150 mm). The initial blood flow rate was set at 150 ml/min. The extracorporeal circuit and filter were primed with 1000 ml of normal saline containing 50 mg of unfractionated heparin before CRRT initiation. The circuit was then flushed with an additional 500 ml of normal saline. The dose was set at 25–30 ml/(kg·h). In the CVVH group, a 1:1 ratio of pre- and post-dilution modes was used. In the CVVHDF group, a post-dilution mode was employed with a 1:1 ratio of replacement fluid to dialysate. A 4% sodium citrate solution was infused at a rate of 200 ml/h from the machine’s pre-pump port for anticoagulation. In the absence of clotting, the filter circuit was routinely replaced at 72 h of CRRT, as recommended by the filter manufacturer. The schematic diagram for the use of RCA-based CRRT with calcium-containing replacement solution in different modalities is shown in Fig. 2.

All patients used the commercial calcium-containing replacement solution (Qingshan Likang, Pharmaceutical Co. Ltd.). This solution consisted of two components: Solution A (containing glucose 10.6 mmol/L, Cl⁻ 118 mmol/L, Mg²⁺ 0.797 mmol/L, Ca²⁺ 1.60 mmol/L, and Na⁺ 113 mmol/L) and Solution B (5% sodium bicarbonate). When combined, 2 L of Solution A and 125 mL of Solution B had a pH of 7.40 and contained glucose 10.0 mmol/L, Cl⁻ 110 mmol/L, Mg²⁺ 0.75 mmol/L, Ca²⁺ 1.50 mmol/L, Na⁺ 141 mmol/L, and HCO₃⁻ 35 mmol/L. During RCA- CRRT, a relatively small dose of 5% sodium bicarbonate (Solution B; 2 L/40 mL) was delivered prefilter by a dedicated pump. This dose could be adjusted at any time to correct metabolic acidosis or alkalosis. Additionally, 10% potassium chloride was infused prefilter by another pump, with the infusion rate and net ultrafiltration adjusted according to blood gas analysis results. 10% calcium gluconate (Qingshan Likang, Pharmaceutical Co. Ltd.) was administered intermittently as an intravenous bolus, as needed, to maintain the target ionized calcium (iCa) level. Acid–base and electrolyte parameters (Ca²⁺, Na⁺, K⁺) were measured at the start of CRRT (prefilter), at 2 h, 6 h, and every 6 h thereafter (both prefilter and postfilter) using the Cobas b 123 system (Roche Diagnostics, Basel, Switzerland). A 4% trisodium citrate solution (Qingshan Likang, Pharmaceutical Co. Ltd.) was infused into the arterial line of the extracorporeal circuit at an initial rate of 200 mL/h, with adjustments made in increments or decrements of 10 mL/h as needed to achieve target ionized calcium levels of 0.25–0.35 mmol/L postfilter and 1.0–1.1mmol/L prefilter.

### Data collection and clinical outcomes

We collected clinical information and laboratory variables at the start and end of CRRT treatment. Arterial blood gas analysis was performed at the start of CRRT and 2 h, 6 h, and every 6 h thereafter in both pre-filter and post-filter blood. Other information during CRRT was also recorded, including catheter site, blood flow rate, infusion rates of 4% sodium citrate and 10% calcium gluconate, filtration fraction (FF). The FF is calculated as: (pre-dilution rate + post-dilution rate + ultrafiltration rate) divided by [blood flow rate × (1 - hematocrit) × 60 + pre-dilution rate], with all rates expressed in mL/h except blood flow in mL/min.

The primary outcomes were extracorporeal circuit lifespan, clotting incidence, and early extracorporeal circuit failure rates during CRRT. Extracorporeal circuit failure was defined as the termination of CRRT because of a transmembrane pressure exceeding 300 mm Hg, a visible blood clot that obstructed blood flow through the filter, or the venous chamber was unable to rotate due to blood clot obstruction. The circuit lifespan was calculated as the duration from CRRT initiation to session cessation, which occurred under the following circumstances: (1) extracorporeal circuit failure, (2) reaching the manufacturer-recommended time limit (72 h), or (3) non-clotting-related cessation (e.g., treatment goal achievement, patient transfer for surgery, CRRT technical malfunctions, or death) [12]. Extracorporeal circuit clotting was categorized into filter clotting and venous chamber clotting. The severity of filter and venous chamber clotting was assessed using a semi-quantitative grading system (Grade 1: mild; Grade 2: moderate; Grade 3: severe), as detailed in Table 3 [13]. Meanwhile, according to previous studies, early circuit failure was defined as lifespan < 48 h [14, 15]. The secondary endpoints included the changes in laboratory parameters such as hemoglobin (Hb), platelets (PLT), hematocrit (Hct), pH, lactate, serum creatinine, urea, total bilirubin, prothrombin time (PT), and activated partial thromboplastin time (APTT) before and after CRRT treatment. Additionally, differences in blood flow rate and sodium citrate infusion rate among the three CRRT modalities, as well as the 30-day all-cause mortality, were also assessed.

### Sample size and randomization

Eligible patients will be randomly assigned to three groups to receive different modalities for the initiation of CRRT at a 1:1:1 ratio, determined by a central computer-generated randomized sequence. Due to practical considerations, blinding of patients and treating clinicians was clinically impractical during this study. The study’s sample size was calculated based on G-Power 3.1.9.2 for analysis of variance (ANOVA) with 80% power and a two-tailed alpha of 0.05. According to previous literature [5, 16], the mean circuit lifespans under citrate anticoagulation were 63.5 ± 27.1 h (CVVH), 55.1 ± 21.8 h (CVVHDF), and 45.6 ± 25.6 h (CVVHD). The estimated minimum required sample size per group was 39. To account for a 10% dropout rate, the adjusted sample size was increased to 40 per group (total *N* = 120).

### Monitoring

To ensure the quality and regulatory compliance of the trial, We established an independent data monitoring committee (DMC) with five members, including experts in nephrology, CRRT management, nursing, trial methodology, and biostatistics. All adverse events were meticulously documented and managed per protocol-specified algorithms, with longitudinal tracking until definitive resolution or clinical stabilization.

### Statistical methods

Continuous variables were expressed as mean with standard deviation if normally distributed, or median with interquartile range (IQR) if non-normally distributed. Categorical variables are reported as counts with percentages. Group comparisons were performed using Fisher’s exact tests or Kruskal-Wallis tests as appropriate. Longitudinal data with multiple time points were analyzed using RM-ANOVA with Greenhouse-Geisser correction, followed by Bonferroni post hoc tests. Kaplan–Meier survival analysis using the log-rank test was implemented to compare circuit lifespan between groups. Statistical significance was assigned to P values < 0.05. All analyses and graphs were performed using R software, version 4.4.1.

## Results

### Baseline characteristics

From June 2022 to May 2023, a total of 467 critically ill AKI patients requiring CRRT were assessed for eligibility, with 328 (70.2%) excluded based on predefined exclusion criteria. Of 139 randomly allocated participants, 18 (12.95%) met post-randomization exclusion criteria: administration of non-protocol anticoagulation (*n* = 8) and other exclusionary events (*n* = 10). Consequently, 121 patients were included in the study: 40 patients in CVVH group, 40 patients in CVVHD, and 41 patients in CVVHDF group. The flowchart of this RCT is illustrated in Fig. 1.

Demographic and clinical characteristics at the time of randomization were similar across the groups and are listed in Table 1. The three groups demonstrated comparable baseline characteristics, including demographic, clinical, and laboratory parameters at CRRT initiation (all *P* > 0.05). At the initiation of CRRT, 57 patients (47.1%) were in AKI stage 2, and 64 patients (52.9%) were in AKI stage 3.

**Table 1:** Characteristics of participants

Variables	CVVH (*n* = 40)	CVVHD (*n* = 40)	CVVHDF (*n* = 41)
Gender			
Males	28 (70.0)	28 (70.0)	30 (73.2)
Age, years, (SD)	58 ± 17	54 ± 20	54 ± 17
BMI, kg/m^2^ (SD)	24.40 ± 4.53	23.33 ± 5.04	23.85 ± 3.63
*Illness severity at* admission *to the ICU*			
SOFA score (SD)	12.18 ± 2.28	12.48 ± 3.14	12.10 ± 2.67
**Mechanical Ventilation**,** n**,** (%)**	37 (92.5)	36 (90)	38 (92.7)
*Etiology*			
**AKI stage** (%)			
Stage 2	19 (47.5)	17 (42.5)	21 (51.2)
Stage 3	21 (52.5)	23 (57.5)	20 (48.8)
Etiology of AKI, n (%)			
Sepsis	28 (70)	27 (67.5)	28 (68.3)
**Hypovolemia**	4 (10)	3 (7.5)	3 (7.3)
**COVID-19**	2 (5)	3 (7.5)	3 (7.3)
**cardiac surgery**	3 (7.5)	5 (12.5)	5 (12.2)
Others	3 (7.5)	2 (5)	2 (4.9)
*Laboratory data at CRRT initiation*			
Hemoglobin, g/L (SD)	83 ± 19	85 ± 22	81 ± 20
Platelet,10^9/L (SD)	99 ± 65	110 ± 70	103 ± 85
Hematocrit (SD)	26 ± 6	27 ± 7	26 ± 6
**Bilirubin**, µmol/L (IQR)	17.9 (9.8,27.1)	15.5 (8.8,21.5)	20.2(12.0,30.9)
Creatinine, µmol/L (IQR)	207 (177,239)	277 (227,410)	233 (185, 319)
Blood Urea Nitrogen, mmol/L (IQR)	14.0 (10.0,18.1)	17.0 (12.1,23.7)	14.9 (9.6,20.8)
Triglycerides, mmol/L (IQR)	1.7 (1.1,2.3)	2.0 (1.2,3.0)	1.6 (1.0,2.9)
**Total Cholesterol**, mmol/L (IQR)	2.5 (1.9,3.3)	2.5 (1.8,3.2)	2.4 (1.8,3.2)
PT, s (IQR)	13.5 (11.9,16.3)	13.1 (11.7,14.5)	13.0 (11.7,14.3)
APTT, s (IQR)	34.0 (30.2,37.6)	31.5 (28.3,40.7)	31.4 (29.8,37.4)
**D-dimer**, mg/L (IQR)	7.2 (3.9,12.3)	5.7 (4.2,12.2)	4.4 (2.7,9.9)
**IL-6**,** ng/ml (IQR)**	204 (108,404)	155 (48,296)	149(66,365)
**PH (SD)**	7.34 ± 0.09	7.35 ± 0.07	7.36 ± 0.06
Lactate, mmol/L **(SD)**	1.84 ± 0.79	1.96 ± 0.73	1.71 ± 0.56
**Magnesium**,** m**mol/L (IQR)	1.02 ± 0.24	0.93 ± 0.14	0.99 ± 0.31
**phosphate**,** m**mol/L (IQR)	1.20 ± 0.43	1.40 ± 0.43	1.35 ± 0.54
**Ionized Calcium**, mmol/L (IQR)	1.06 ± 0.11	1.01 ± 0.16	1.03 ± 0.08

CVVH, continuous veno-venous hemofiltration; CVVHD, continuous veno-venous hemodialysis; CVVHDF, continuous veno-venous hemodiafiltration; SD, standard deviation; IQR, interquartile range; SOFA, sequential organ failure assessment; AKI: Acute Kidney Injury; CRRT: continuous renal replacement therapy; BMI: Body Mass Index; PT: Prothrombin Time; APTT: Activated Partial Thromboplastin Time; Interleukin-6:IL-6;PH: Potential of Hydrogen

### Treatment parameters during CRRT

Treatment parameters during CRRT were summarized in Table 2. For vascular access, the right femoral vein was used most frequently (56.2%), followed by the left femoral vein (33.1%) and right internal jugular vein (10.7%); the left internal jugular vein was not used. Significant variations in FF were observed among the different CRRT modalities (*P* < 0.001). CVVH demonstrated the highest median FF (33.1%, IQR 32.4–35.7), significantly exceeding both CVVHDF (19.1%, IQR 17.7–20.7) and CVVHD (4.0%, IQR 3.6–4.9). Blood flow rates (141 ± 8 ml/min vs. 139 ± 9 ml/min vs. 141 ± 8 ml/min) and 4% trisodium citrate infusion rates (195 ± 6 ml/h vs. 209 ± 2 ml/h vs. 195 ± 5 mL/h) were adjusted to maintain postfilter iCa levels within 0.25 to 0.35 mmol/L in CVVH, CVVHD, and CVVHDF groups, respectively.

**Table 2:** Treatment parameters of the patients with AKI receiving CRRT

	CVVH (*n* = 40)	CVVHD (*n* = 40)	CVVHDF (*n* = 41)	*p* value
*Vascular access*, *n*, (%)				
Left femoral	16 (40)	9 (22.5)	15 (36.6)	0.539
Right femoral	20 (50)	26 (65)	22 (53.7)	
Internal jugular	4 (10)	5 (12.5)	4 (9.7)	
Blood flow rate, ml/min (SD)	141 ± 8	139 ± 9	141 ± 8	0.635
Filtration fraction, % (IQR)	33.1 (32.4,35.7)	4.0 (3.6,4.9)	19.1 (17.7,20.7)	< 0.001
4% trisodium citrate infusion rate, ml/h (SD)	195 ± 6	209 ± 2	195 ± 5	< 0.001

CVVH, continuous veno-venous hemofiltration; CVVHD, continuous veno-venous hemodialysis; CVVHDF, continuous veno-venous hemodiafiltration; SD, standard deviation; IQR, interquartile range

### Primary outcomes

**Fig. 3:**
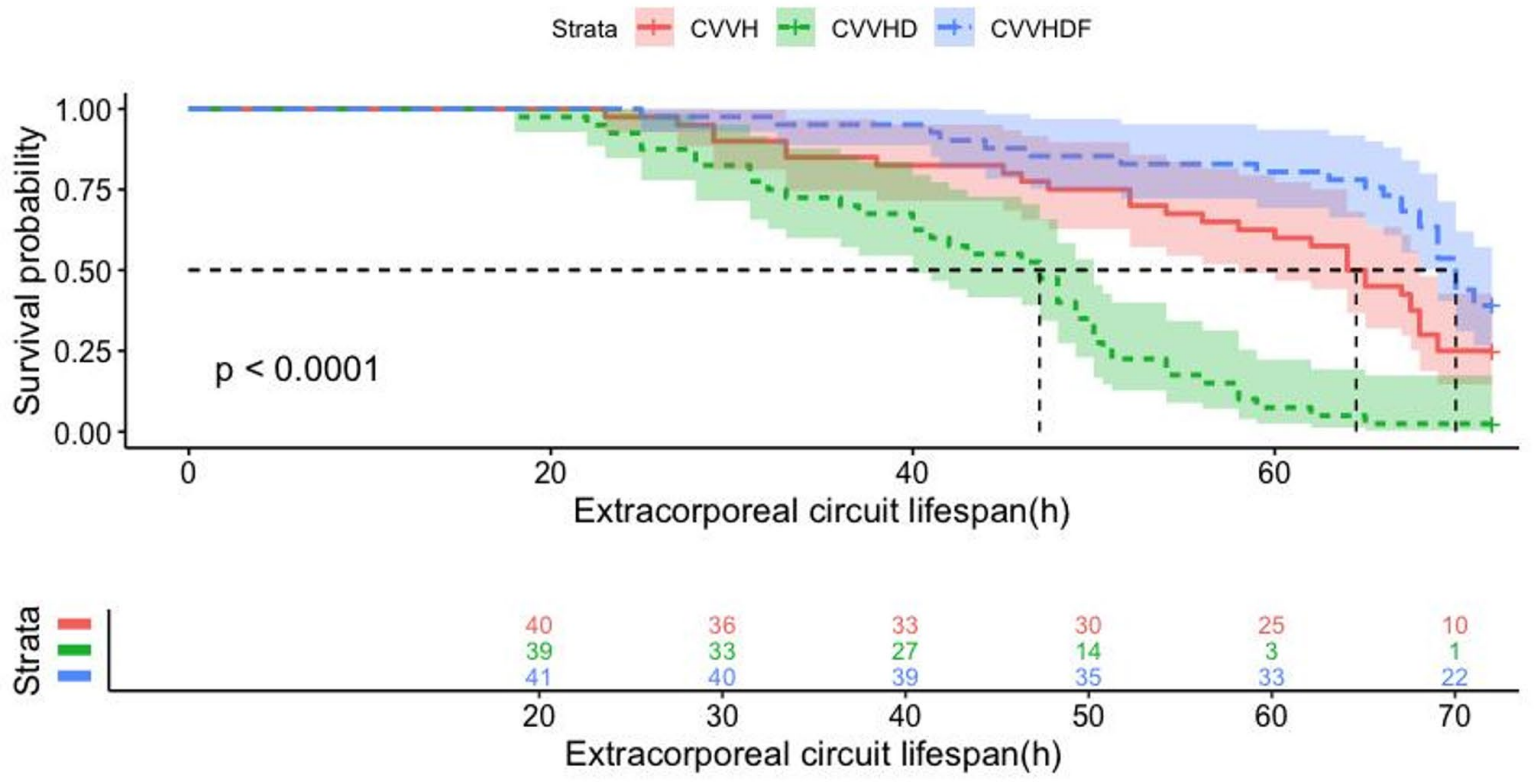
Kaplan–Meier survival curve for extracorporeal circuit lifespan of per circuit. CVVH, continuous veno-venous hemofiltration; CVVHD, continuous veno-venous hemodialysis; CVVHDF, continuous veno-venous hemodiafiltration

The results of primary outcomes were presented in Table 3. The median circuit lifespan in all circuits was 60 h (IQR 44.5, 70). CVVHDF group demonstrated a significantly longer median circuit lifespan (70 h, IQR 65–72) compared to both CVVH (64 h, IQR 46–71) and CVVHD group (47 h, IQR 31.5–54) (*P* < 0.001). Intergroup comparisons demonstrated statistically significant differences in extracorporeal circuit lifespan (CVVH vs. CVVHD: *P* = 0.004; CVVH vs. CVVHDF: *P* = 0.014; CVVHD vs. CVVHDF: *P* < 0.001). Consistent results were also demonstrated by the Kaplan–Meier curve and log-rank test for extracorporeal circuit lifespan among groups in Fig. 3. Extracorporeal circuit clotting is a critical factor influencing lifespan. In our study, circuits remained patent without clotting for the full 72-h duration in 27 circuits (22.3%). Among 94 circuits with extracorporeal circuit thrombosis, severe venous chamber clotting resulted in CRRT termination in 23 circuits (24.5%). 94 circuits were terminated due to severe clotting, comprising 71 filter clotting events (75.5%) and 23 venous chamber clotting events (24.5%). The CVVHD group demonstrated a markedly higher rate of severe venous chamber thrombosis (50.0%, 20/40) compared to both CVVH (5.0%, 2/40) and CVVHDF (2.4%, 1/41) groups (*P* < 0.001), with no significant difference observed between CVVH and CVVHDF (*P* = 0.616). The details for clotting in each group are presented in Table 3. Among the three groups, a total of 35 circuits (28.9%) experienced early failure of extracorporeal circulation (< 48 h). Specifically, there were 9 circuits (22.5%) in the CVVH group, 21 circuits (52.5%) in the CVVHD group, and 5 circuits (12.2%) in the CVVHDF group (*P* = 0.004).

**Table 3:** Circuits and clinical outcomes of patients

	CVVH (*n* = 40)	CVVHD (*n* = 40)	CVVHDF (*n* = 41)	*p* value
Circuit lifespan (IQR)	64 (46,71)	47 (31.5,54)	70 (65,72)	< 0.001
Incidence of circuits remained patent for 72 h	10 (25%)	1 (2.5%)	16 (39%)	0.03
Incidence of early failure *n*/total (%)	9 (22.5%)	21 (52.5%)	5 (12.2%)	0.04
*Clotting sites and grading system*				
Filter clotting *n*/total (%)				
1 (*presence of fibrin*)	2 (5.0)	4 (10.0)	5 (12.2)	< 0.001
2 (*presence of small blood clot*)	10 (25.0)	17 (42.5)	12 (29.3)	
3 (*visible clot occupying > 50% of filter volume accompanied by significant elevation in transmembrane pressure*)	28 (70.0)	19 (47.5)	24 (58.5)	
Venous chamber clotting *n*/total (%)				
1 (presence of fibrin)	12 (30.0)	4 (10.0)	20 (48.8)	< 0.001
2 (presence of small blood clot)	23 (57.5)	16 (40.0)	14 (34.2)	
3 (presence of blood clot *accompanied by significant elevation in venous pressure*)	2 (5.0)	20 (50.0)	1 (2.4)	
Outcome of Patients 30-d mortality *n*/total (%)	8 (20%)	7 (17.5%)	7 (17.1%)	0.957
*Adverse events n*/total (%)				
Citrate accumulation	3 (7.5%)	2 (5.0%)	3 (7.3%)	0.881
Bleeding	1 (2.5%)	0 (0%)	1 (2.5%)	0.605

CVVH, continuous veno-venous hemofiltration; CVVHD, continuous veno-venous hemodialysis; CVVHDF, continuous veno-venous hemodiafiltration; IQR, interquartile range

### Efficacy and safety of RCA-CRRT using calcium-containing replacement solution

Pre-CRRT median blood urea levels differed across CVVH (14.0 mmol/L, IQR 10.0-18.1), CVVHD 17.0 mmol/L, IQR 12.1–23.7), and CVVHDF (14.9 mmol/L, IQR 9.6–20.8) groups, with significant post-treatment reductions to 9.9 mmol/L (IQR 5.6–12.6), 11.7 mmol/L (IQR 7.6–16.0), and 8.4 mmol/L (IQR 6.4–14.6) respectively (*P* < 0.001). Similar significant decreases occurred in creatinine levels (*P* < 0.001). Inflammatory markers showed modality-dependent responses: Procalcitonin declined significantly across all groups (*P* < 0.01), while IL-6 reduction reached significance only in CVVH (204 pg/mL [IQR 108–404] to 81 pg/mL [IQR 39–240], *P* < 0.05). Significant reductions in serum magnesium and phosphorus levels in post-CRRT (*P* < 0.05), no cases of hypomagnesemia (< 0.7 mmol/L) or hypophosphatemia (< 0.8 mmol/L) were observed clinically. Meanwhile, hemoglobin, coagulation parameters (e.g., PT, APTT, INR), and bilirubin remained unchanged. Changes in Hematologic markers among the three groups of patients are shown in Supplementary Table 1.

30-day survival analysis demonstrated comparable all-cause mortality across treatment groups (*P* = 0.957), with rates of 20.0% (8/40) for CVVH, 17.5% (7/40) for CVVHD, and 17.1% (7/41) for CVVHDF group. Among the 121 patients, 8 (6.61%) patients had citrate accumulation, with 3 (7.50%) in the CVVH group, 2 (5.00%) in the CVVHD group, and 3 (7.32%) in the CVVHDF group (*P* = 0.881). Citrate accumulation was resolved by temporarily increasing calcium gluconate and pausing citrate. 2 patients (1.65%) had bleeding complications, one in the CVVH group with tracheostomy site oozing and one in the CVVHDF group with gastrointestinal bleeding (*P* = 0.605). No patient experienced hyponatremia, arrhythmia or metabolic alkalosis.

## Discussion

### Key findings

In this open-label, parallel-group randomized controlled trial, our results demonstrated that a simplified RCA protocol utilizing calcium-containing replacement solution maintained favorable filter longevity and safety profiles across multiple groups, indicating its potential for clinical application in CRRT. Importantly, CVVHDF may emerged as the optimal modality in our simplified RCA-CRRT protocol. Compared with CVVH and CVVHD, CVVHDF tended to superior circuit survival, lower rates of severe venous chamber thrombosis and early circuit failure, and a comparable incidence of adverse events under this simplified RCA-CRRT protocol.

### Comparison with previous studies

In the management of CRRT for critically ill AKI patients, RCA proved clinically superior to heparin-based strategies, providing both extended extracorporeal circuit survival and significantly fewer bleeding events [17, 18]. Traditional RCA-CRRT typically employs calcium-free replacement fluid, necessitating additional intravenous calcium supplementation (e.g., calcium chloride or calcium gluconate) to maintain systemic ionized calcium (iCa²⁺) levels. However, this approach is operationally complex and carries metabolic risks, such as hypocalcemia and citrate accumulation, as insufficient calcium supplementation may lead to premature circuit clotting. In contrast, RCA-CRRT using calcium-containing replacement fluid [5–7] reduces the need for exogenous calcium infusion, simplifies nursing procedures, and has a comparable extracorporeal circuit lifespan, making simplified RCA-CRRT an important anticoagulation strategy. Meanwhile, Our study further demonstrated that citrate anticoagulation with calcium-containing replacement solution achieved median circuit survival of 60 h of 60 h (IQR 44.5–70) during CRRT, thereby validating the efficacy of this simplified citrate protocol. For CRRT, the treatment modality also significantly impacts the lifespan of the extracorporeal circuit [19]. However, there are currently no RCT comparing the effects of different modalities (CVVH, CVVHD, CVVHDF) in simplified RCA-CRRT on circuit survival.

CRRT solute clearance mechanisms include diffusion, convection, and adsorption. Specifically, CVVH mainly utilizes convection, CVVHD predominantly depends on diffusion, and CVVHDF combines both diffusion and convective processes. Convection-predominant CVVH demonstrates enhanced protein adsorption and middle/large molecule clearance that may occlude membrane pores, increasing clotting risk. In contrast, diffusion-based CVVHD maintains superior hemodynamic stability with minimal hemoconcentration, stable hematocrit, lower transmembrane pressure, and reduced FF- all theoretically favoring extended circuit longevity. Importantly, convective transport promotes prothrombotic effects through multiple pathways: Klingel et al. demonstrated elevated D-dimer and thrombin-antithrombin III complexes [20], while equivalent blood flows generate higher shear stress-mediated platelet activation in convective modes [21]. These mechanistic differences underscore the critical balance between clearance efficiency and circuit survival in modality selection. Ricci et al. conducted a prospective crossover study comparing extracorporeal circuit longevity between CVVH and CVVHD modalities (15 circuits per modality), demonstrating significantly prolonged circuit survival with CVVHD (37 h [IQR 19.5–72.5] vs. 19 h [IQR 12.5–28], *P* = 0.03) [22]. However, the small sample size may limit the generalizability of these findings due to potential selection bias. A post hoc multivariate analysis of a multicenter RCT suggested that CRRT modality was significantly associated with extracorporeal circuit lifespan [3]. Specifically, CVVHD had the longest lifespan. CVVHDF had the second-longest lifespan, with a mean difference of 9.54 h compared to CVVH (95% CI, 0.23–18.85 h; *P* = 0.045). However, this study did not separately compare RCA and heparin anticoagulation when assessing circuit lifespan across different CRRT modalities. In this study, the overall extracorporeal circuit Survival for RCA-CRRT was 46.5 (18.8–70.3) h, whereas in our study, the overall circuit life was 60 h (IQR 44.5, 70), which is significantly longer. Although several studies showed that CVVHD had the longest circuit survival during CRRT, a study compared 197 CVVHD filters and 97 CVVH filters in 39 patients and found no significant difference in circuit lifespan between the two group [23].

In our study, under citrate anticoagulation with calcium-containing replacement fluid, the CVVHDF group had the longest extracorporeal circuit life at 70 h (IQR 65, 72), followed by the CVVH group at 64 h (IQR 46, 71), while the CVVHD group had the shortest at 47 h (IQR 31.5, 54) (*P* < 0.001). Clinical evidence supports maintaining FF ≤ 20–25%, as elevated values induce hemoconcentration (increased post-filter hematocrit), thereby elevating thrombotic risk and impairing filter longevity [24]. In our work, severe filter clotting incidence was highest in CVVH (28/40, 70.0%) group, followed by CVVHDF (24/41, 58.5%), and lowest in CVVHD (19/40, 47.5%) group (*P* < 0.001), consistent with the observed FF gradients across modalities. Meanwhile, the FF was significantly higher in CVVH compared to CVVHDF group (33.1%, IQR 32.4–35.7 vs. 19.1%, IQR 17.7–20.7, *P* < 0.001), which might explain the shorter circuit survival time observed in the CVVH group. Contrary to conventional expectations, the CVVHD group, despite having the lowest FF among all modalities, showed a significantly higher prevalence of severe venous chamber thrombosis(20/40, 50%). This phenomenon substantially reduced extracorporeal circuit longevity in the CVVHD group.

Our findings differ from previous reports due to two key factors: venous chamber thrombosis and device-specific design of the venous chamber. Venous chamber (in addition to the filter) is a critical site of thrombosis in the CRRT extracorporeal circuit. Prior studies indicated 23% of CRRT circuit failures originate from venous chamber clotting [25], likely initiated by microclots dislodged from filters. Additionally, bubble formation from heated replacement fluids or improper sampling [26, 27] exacerbates clotting at blood-air interfaces. Venous reservoirs can be categorized into vertical and horizontal blood inlet types based on the blood entry location. The Prismaflex machine’s horizontal-inlet venous chamber design (blood inlet inferior, replacement fluid superior) creates a protective fluid layer that minimizes blood-air contact. In our study, for CVVH mode, we used a 1:1 ratio of pre- to post-replacement fluid, and for CVVHDF mode, we used only post-replacement fluid with a 1:1 ratio of dialysate to post-replacement fluid. This approach created a protective liquid layer on the blood surface in the venous reservoir for both CVVH and CVVHDF modes. In contrast, CVVHD group’s absence of replacement fluid created direct blood-air interfaces, significantly increasing clotting risk. In our study, among 23 cases of circuit failure due to severe venous chamber thrombosis, CVVHD group accounted for 86.96% (20/23), significantly higher than CVVH (2/23, 8.69%) and CVVHDF (1/23, 4.35%) (*P* < 0.001). These two groups exhibited significantly lower rates of venous chamber thrombosis due to the formation of protective interfacial layers created by replacement fluid infusion. Our finding starkly contrasts with the report by Baldwin et al. [28], which documented much shorter circuit longevity: 13.9 ± 9.5 h for vertical-inlet designs and 17.7 ± 15.9 h for horizontal-inlet designs. Notably, our cohort achieved a superior median circuit survival of 60 h (IQR 44.5–70) compared to previous studies. Investigating the impact of CVVH and CVVHDF modes on the extracorporeal circuit life of RCA-CRRT is also crucial. A retrospective study of 80 pediatric patients receiving CRRT demonstrated significantly prolonged circuit longevity with CVVHDF compared to CVVH (median value [interquartile range]; 47 [15] vs. 35 [17.5] h, *P* = 0.029) [29]. A retrospective cohort study including 182 circuits demonstrated that CVVHDF significantly extended circuit longevity compared to alternative modalities during CRRT [30]. These findings were corroborated by a meta-analysis of 1,143 patients, which revealed that CVVHDF was associated with a significantly longer extracorporeal circuit lifespan than CVVH [31]. Our findings indicated significantly prolonged median circuit survival with CVVHDF versus CVVH (70 h [IQR 65–72] vs. 64 h [IQR 46–71]; *P* = 0.023) when using the Prismaflex machine with simplified RCA-CRRT.

In our work, while CRRT modalities showed significant differences in circuit longevity (*P* < 0.01), 30-day mortality did not differ (*P* = 0.957). This suggested that: (1) CRRT technical factors might have been outweighed by baseline disease severity and other confounders; (2) proactive circuit management (e.g., timely replacements) may have mitigated the impact of technical variations on clinical outcomes. Meanwhile, the mortality rate in our cohort was markedly lower compared to the RCA group (41%, 183/443) in prior meta-analytic data [32].

In terms of efficacy and safety, all modalities achieved significant reductions in creatinine and blood urea nitrogen (*P* < 0.001) without affecting hematologic parameters (e.g. Hb, PLT, INR, and APTT) during CRRT compared to pre-treatment levels in our study (shown in STab1). However, another study showed significant decreases in Hb (*P* = 0.017), PLT (*P* = 0.043), and INR (*P* = 0.018) during 72 h of CRRT, while systemic APTT showed a non-significant downward trend (*P* = 0.077) [33]. Concerning adverse events, citrate accumulation rates demonstrated no significant differences across three groups (Table 3), aligning with previously reported frequencies in previous studies [3, 34]. Only one patient of bleeding occurred in each of the CVVH (1/40) and CVVHDF (1/41) groups. No other adverse complications were observed in the three group.

So we employed a randomized controlled design to assess the safety and efficacy of different modalities during RCA-CRRT with calcium-containing replacement solution. Our study incorporated a substantial sample size, enabling a comprehensive assessment of circuit performance and patient outcomes across CVVHDF and other modalities (CVVH, CVVHD) during CRRT. These findings might have contributed to building an evidence-based foundation for considering optimal modalities in simplified RCA-CRRT therapy.

### Limitation

Several limitations of this study should be acknowledged. First, the open-label design may introduce performance and detection bias. Second, the absence of long-term outcome data, including renal recovery (e.g., 90-day dialysis-free survival), ICU and hospital length of stay, limited our ability to assess the sustained clinical benefits of the interventions. Third, as all CRRT machines used were Prismaflex models, the generalizability of our results to other devices remains uncertain due to differences in venous chamber designs. Additionally, some patients had received RCA-CRRT prior to enrollment (though new extracorporeal circuits were used after inclusion), which may have introduced confounding effects. Furthermore, this trial was conducted at a single tertiary care center in Southwest China. Future multicenter studies with larger sample sizes are needed to validate these findings.

## Conclusions

In our study, the use of RCA with calcium-containing replacement solution during CRRT appeared to be effective and safe, based on the observed outcomes. Compared to CVVH and CVVHD, CVVHDF might be preferable for simplified RCA-CRRT, as they exhibited a longer circuit survival and lower incidence of severe venous chamber thrombosis without an increase in the risk of adverse events. Further high-quality, multicenter RCTs are essential to reinforce the evidence guiding modality choice during CRRT.

## Supplementary Information

Below is the link to the electronic supplementary material.

Supplementary Material 1

## Data Availability

The datasets used and/or analyzed during the current study are available from the corresponding author on reasonable request.
