# Supplementary material for: Efficacy and safety of regional citrate anticoagulation using calcium-containing replacement solution in different modalities of continuous renal replacement therapy: a randomized controlled trial
Source: BMC Nephrol. 2025 Nov 6;26:624. doi: 10.1186/s12882-025-04565-7 (PMC12590661; doi:10.1186/s12882-025-04565-7)
Supplement: Supplementary file 1 — Supplementary Material 1 [file 12882_2025_4565_MOESM1_ESM.docx]

**Table S1. Changes in Laboratory Parameters among the three groups of patients during CRRT**

**Comparison of Laboratory Parameters Before and After CRRT Among Three Treatment Groups**

| Variables | Time | CVVH  (n=40) | CVVHD  (n=40) | CVVHDF  (n=41) | *P value* |
| --- | --- | --- | --- | --- | --- |
| Blood Urea Nitrogen, mmol/L (IQR) | pre-CRRT | 14.0（10.0,18.1） | 17.0（12.1,23.7） | 14.9（9.6,20.8） | 0.422 |
|  | post-CRRT | 9.9（5.6,12.6）^***^ | 11.7（7.6,16.0）^***^ | 8.4（6.4,14.6）^***^ | 0.496 |
| Creatinine, µmol/L (IQR) | pre-CRRT | 207（177,239） | 277（227,410） | 233（185,319） | 0.104 |
|  | post-CRRT | 113（81,172）^***^ | 157（75,235）^***^ | 113（76,182）^***^ | 0.377 |
| Hb, g/L (SD) | pre-CRRT | 83±19 | 85±22 | 81±20 | 0.654 |
|  | post-CRRT | 81±13 | 81±16 | 81±14 | 0.982 |
| PLT, 10^9^/L (SD) | pre-CRRT | 99±65 | 110±70 | 103±85 | 0.946 |
|  | post-CRRT | 101±54 | 110±85 | 121±98 | 0.628 |
| Hematocrit (SD) | pre-CRRT | 0.26±0.06 | 0.27±0.07 | 0.26±0.06 | 0.773 |
|  | post-CRRT | 0.26±0.04 | 0.25±0.05 | 0.26±0.05 | 0.792 |
| INR, (SD) | pre-CRRT | 1.13±0.30 | 1.21±0.23 | 1.30±0.55 | 0.615 |
|  | post-CRRT | 1.27±0.28 | 1.29±0.41 | 1.28±0.49 | 0.806 |
| PT, s (IQR) | pre-CRRT | 13.5（11.9,16.3） | 13.1（11.7,14.5） | 13.0（11.7,14.3） | 0.887 |
|  | post-CRRT | 13.9（11.6,15.7） | 12.9（11.9,14.9） | 12.8（11.5,13.9） | 0.615 |
| APTT, s (IQR) | pre-CRRT | 34.0（30.2,37.6） | 32.3（28.6,34.2） | 34.2（29.2,37.4） | 0.506 |
|  | post-CRRT | 31.7（28.2,37.6） | 31.5（28.3,40.7） | 31.4（29.8,37.4） | 0.628 |
| **Potassium**,  mmol/L (SD) | pre-CRRT | 4.48±0.66 | 4.51±0.51 | 4.54±0.60 | 0.934 |
|  | post-CRRT | 4.19±0.31 | 4.27±0.26 | 4.26±0.31 | 0.125 |
| **Phosphate, m**mol/L (IQR) | pre-CRRT | 1.20±0.43 | 1.40±0.43 | 1.35±0.54 | 0.743 |
|  | post-CRRT | 0.92±0.37^*^ | 0.93±0.40^***^ | 0.89±0.36^**^ | 0.769 |
| **Magnesium, m**mol/L (IQR) | pre-CRRT | 1.02±0.24 | 0.93±0.14 | 0.99±0.31 | 0.300 |
|  | post-CRRT | 0.88±0.13^***^ | 0.90±0.12^*^ | 0.86±0.10^**^ | 0.319 |
| Procalcitonin, ng/ml | pre-CRRT | 5.7（1.5,11.0） | 4.0（1.1,15.3） | 2.3（1.2,7.2） | 0.272 |
|  | post-CRRT | 2.5（1.1,4.8）^***^ | 1.4（0.6,5.5）^***^ | 1.5（0.9,4.8）^**^ | 0.635 |
| IL-6, pg/ml | pre-CRRT | 204（108,404） | 155（48,296） | 149（66,365） | 0.069 |
|  | post-CRRT | 81（39,240）^*^ | 56（36,170） | 66（24,179） | 0.671 |

INR: International Normalized Ratio; PT: Prothrombin Time; APTT: Activated Partial Thromboplastin Time;

*Within-group comparison vs. pre-CRRT: P < 0.05, P < 0.01, **P < 0.001
